# Delta Radiomics Model Predicts Lesion-Level Responses to Tyrosine Kinase Inhibitors in Patients with Advanced Renal Cell Carcinoma: A Preliminary Result

**DOI:** 10.3390/jcm12041301

**Published:** 2023-02-06

**Authors:** Yuntian Chen, Enyu Yuan, Guangxi Sun, Bin Song, Jin Yao

**Affiliations:** 1 Department of Radiology, West China Hospital, Sichuan University, Chengdu 610000, China; 2 Department of Urology, West China Hospital, Sichuan University, Chengdu 610017, China; 3 Department of Radiology, Sanya People’s Hospital, Sanya 572000, China

**Keywords:** radiomics, renal cell carcinoma, tyrosine kinase inhibitor, molecular targeted therapy, treatment response

## Abstract

Background: This study aimed to develop and internally validate computed tomography (CT)-based radiomic models to predict the lesion-level short-term response to tyrosine kinase inhibitors (TKIs) in patients with advanced renal cell carcinoma (RCC). Methods: This retrospective study included consecutive patients with RCC that were treated using TKIs as the first-line treatment. Radiomic features were extracted from noncontrast (NC) and arterial-phase (AP) CT images. The model performance was assessed using the area under the receiver operating characteristic curve (AUC), calibration curve, and decision curve analysis (DCA). Results: A total of 36 patients with 131 measurable lesions were enrolled (training: validation = 91: 40). The model with five delta features achieved the best discrimination capability with AUC values of 0.940 (95% CI, 0.890‒0.990) in the training cohort and 0.916 (95% CI, 0.828‒1.000) in the validation cohort. Only the delta model was well calibrated. The DCA showed that the net benefit of the delta model was greater than that of the other radiomic models, as well as that of the treat-all and treat-none criteria. Conclusions: Models based on CT delta radiomic features may help predict the short-term response to TKIs in patients with advanced RCC and aid in lesion stratification for potential treatments.

## 1. Introduction

Vascular endothelial growth factor (VEGF) has a well-characterized role in angiogenesis, which is critical in the carcinogenesis and the pathophysiology of renal cell carcinoma (RCC) [1]. Leveraging the molecular mechanism of angiogenesis, targeted therapies against the VEGF pathway have become the first-line treatments for advanced or metastatic RCC (mRCC), of which treatment with tyrosine kinase inhibitors (TKIs) has significantly improved the survival of patients with mRCC in the past decade [2].

Despite the use of clinical prognostic models, such as the International Metastatic Renal Cell Carcinoma Database Consortium (IMDC) score, radiomics has been used to identify patient subgroups responsive to TKI treatment. In previous studies, the features of each lesion and the average values of each feature were used to predict patient-level responses. In a study by Haider et al., size-normalized standard deviation in baseline computed tomography (CT) images was determined to be a significant predictor of progression-free survival (PFS) and to improve the prognostic performance of IMDC [3]. In addition, Goh et al. found that the percentage change in coarse uniformity between the baseline CT and the CT after two treatment cycles independently predicted the time to progression [4]. Notably, the features used in previous studies were derived from a single phase. Considering the mechanism of TKIs and the clinical practice of radiologists, the difference between arterial-phase (AP) features and noncontrast (NC) features, namely delta features, reflects the vascularization or perfusion of the lesion and thus may better predict the response to anti-VEGF-based TKIs.

Not all lesions benefit from TKI treatment. Recent studies have reported heterogeneous radiological responses [5,6]. The term refers to the fact that the responses of the lesions in one patient fall into at least two response categories which reflect different drug sensitivity between lesions [5]. Response Evaluation Criteria In Solid Tumors (RECIST) is commonly applied for response assessment in metastatic cancers. However, in cases of treatment discontinuation owing to RECIST-defined disease progression, a number of lesions may remain controlled. In addition, the occurrence of small new lesions may require switching to alternative therapies, even if several large lesions remain controlled [7]. This necessitates studies on the radiomic assessment of the lesion-level response to TKIs and on the stratification of lesions with different progression risks. Yet, published studies on these aspects are still scant.

In this preliminary study, we aimed to develop and internally validate machine-learning models using radiomics features extracted from pretreatment CT images to predict the lesion-level short-term response to TKIs in patients with advanced RCC. Simultaneously, we aimed to investigate the predictive value of delta radiomics features, which are calculated by subtracting NC CT radiomics features from AP CT radiomics features.

## 2. Materials and Methods

The institutional review board of our institution approved this study, and the requirement for written informed consent was waived for this retrospective analysis.

### 2.1. Study Population

**Figure 1:**
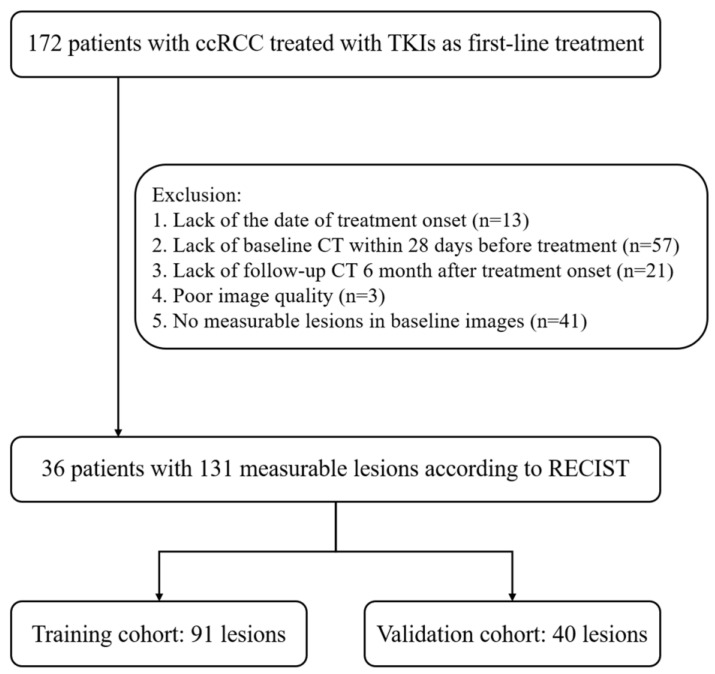
Flowchart for selecting the study population.

This retrospective study screened consecutive patients with RCC treated using TKIs as their first-line treatment between January 2008 and October 2021 at our institution. Patients were included if they met all of the following criteria: (a) patients who had pathologically proven recurrent or metastatic clear-cell RCC and (b) patients who were treated with sunitinib, axitinib, sorafenib, or pazopanib as their first-line treatment. Patients were excluded if they met any of the following criteria: (a) patients with previous systemic therapy, (b) patients who did not complete the treatment cycle (for example, financial burden or side effects), (c) patients without necessary clinical data (for example, date of treatment onset), (d) patients without baseline CT or follow-up CT images, (e) patients with poor image quality, or (f) patients with no measurable lesions on baseline NC or AP CT images. Finally, 36 patients with 131 measurable lesions were enrolled in our study (Figure 1). All images and electronic clinical information were deidentified before being transferred to the investigators.

### 2.2. CT Image Acquisition and Preprocessing

The baseline and follow-up CT images of the chest, abdomen, and pelvis were performed using 128-slice Siemens SOMATOM Force, 256-slice Siemens Definition Flash, 256-slice GE Revolution Apex, or 320-slice UNITED IMAGING UCT960 with the following parameters: tube voltage, 120 keV; tube current, 210 mAs; collimation, 1 mm; and slice thickness, 2 mm. AP images were obtained 30s after an intravenous bolus injection of the contrast agent (Omnipaque 300, GE Healthcare, Shanghai, China) at a concentration of 350 mg/mL and a rate of 1.5 mL/s using a high-pressure pump syringe. The CT images were resampled with a 5 mm slice thickness.

### 2.3. Lesion Identification and Outcome Analysis

The baseline image was defined as the CT image obtained within 28 days before treatment onset, and the follow-up image was defined as the CT image obtained six months after treatment onset. In this study, one radiologist, specialized in abdominal imaging, analyzed all RECIST-defined measurable lesions. Specifically, tumorous lesions with their longest diameter larger than 10 mm and lymph node lesions with a short axis larger than 15 mm were included. First, patient-level responses were evaluated according to the (Response Evaluation Criteria in Solid Tumours) RECIST v 1.1. The presence of new lesions was considered as a progressive disease at the patient level. Second, based on the relative size change compared with that at baseline, each lesion was classified into one of three response categories [5]: responding lesions (RL), characterized by a decrease in size of ≥30%; progressing lesions (PL), characterized by an increase in size of ≥20%; or stable lesions (SL), which did not comply with any of the above two criteria. PLs were considered positive samples, while SLs and RLs were considered negative samples. Furthermore, if the responses of the lesions in one patient fell into at least two response categories, lesion-level responses were considered heterogeneous.

### 2.4. Image Segmentation

The volumes of interest (VOIs) of the included lesions were segmented on axial NC and AP CT images using the ITK-SNAP software (v3.8.0, http://www.itksnap.org accessed on 21 December 2022). VOIs involving the gross volume were delineated in the lung (level, −500 HU; width, 1750 HU) or soft tissue (level, 40 HU; width, 400 HU) window settings according to their locations. One radiologist, who had two years of experience and was not included in the outcome analysis, independently segmented all lesions without any knowledge of the patients’ clinicopathological data and lesion outcomes. To test the intrareader reliability of segmentation, the procedure was repeated by the same radiologist on 30 randomly selected lesions. Two sets of radiomics features were extracted and compared using the intraclass correlation coefficient (ICC).

### 2.5. Feature Extraction

**Figure 2:**
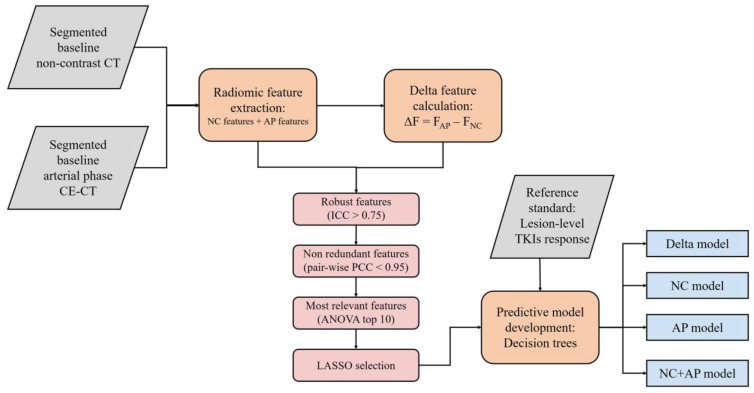
Workflow of radiomic analysis.

The workflow of radiomic analysis is demonstrated in Figure 2. For each phase, 107 radiomics features were extracted. These features were consistent with the Image Biomarker Standardization Initiative [8]. A total of 93 delta radiomics features were computed as the arithmetic difference between the NC and AP features. Because tumor shape was consistent during the CT scans, shape-based delta features were not calculated (*n* = 14). Detailed information regarding the extracted radiomic features can be found in the online documentation (https://pyradiomics.readthedocs.io/en/stable/features.html accessed on 21 December 2022).

### 2.6. Feature Dimensional Reduction and Selection

First, features with low reproducibility (ICC < 0.75) were excluded. Second, the included features were normalized using Z-score standardization. Pairwise Pearson’s correlation analysis was used to remove redundant features. For highly correlated features (Pearson’s correlation coefficient > 0.95), the feature with a higher average absolute correlation was removed. Subsequently, the included features were ranked using the F-value of analysis of variance. The F-value estimates the degree of linear dependency between the features and the outcome to be predicted. Features with the top 10 F-values were entered in the least absolute shrinkage and selection operator (LASSO) regression, and non-zero-weighted features were selected for the final models. The hyperparameter lambda value in LASSO was determined by five-fold cross -validation using the training data to minimize the mean square error (MSE).

### 2.7. Modeling and Evaluation

Four models were developed: (1) the NC model using NC features, (2) the AP model using AP features, (3) the NC + AP model using both NC and AP features, and (4) the delta model using delta features. Using stratified sampling, the lesions were divided into training and validation cohorts at a ratio of 7:3. Decision tree models were trained to classify the samples. To avoid overfitting, the maximum depths of the decision tree models were set to 3. The optimal cut-off values were identified to attain the highest sensitivity while maintaining a specificity of >0.8 in the training cohort. The area under the receiver operating characteristic (ROC) curve (AUC) was used to assess the discrimination ability of the models. Calibration curve analysis was used to graphically report the calibration ability of the models. The sensitivity, specificity, positive predictive value (PPV), negative predictive value (NPV), and accuracy were used to measure the classification performance of the models. Decision curve analysis (DCA) was used to describe and compare the clinical effects of the models. Feature extraction, feature selection, modeling, and validation were implemented using pyradiomics (v3.0), SciPy (v1.8.0), and scikit-learn (v1.1.1) in Python (v3.7.9, https://www.python.org accessed on 21 December 2022).

### 2.8. Statistical Analysis

Descriptive analyses were performed to describe the demographic data characteristics. Variables were compared between the training and validation cohorts using Student’s *t*-test or the Mann–Whitney U test for continuous variables, the chi-square test or Fisher’s exact test for unordered categorical variables, and the Mann–Whitney U test for ordered categorical variables. Delong’s test was used to compare the ROC curves between the models. Statistical significance was set at *p* < 0.05. Delong’s test was performed with pROC (v1.18.0) in R (v4.2.1, https://www.r-project.org accessed on 21 December 2022), and other statistical analyses were performed using SciPy (v1.8.0) and pyirr (v0.84.1.1) in Python.

## 3. Results

### 3.1. Patient Characteristics

The clinical characteristics of the patients are summarized in Table 1. According to RECIST, at six months, 14 patients (38.9%) achieved partial response (PR), 14 patients (38.9%) had stable disease (SD), and 8 patients (22.2%) had progressive disease (PD). Heterogeneous or mixed responses were observed in 13 of 36 patients (36.1%), including 6 of the 14 patients showing PR (42.9%), 3 of the 14 patients having SD (21.4%), and 4 patients having PD (50%).

### 3.2. Outcome Evaluation

The lesions were separated as follows: training cohort (74 RLs/SLs and 17 PLs; 70%) and validation cohort (33 RLs/SLs and 7 PLs; 30%). The characteristics of the lesions are shown in Table 2. The results indicated no significant differences between the training and validation cohorts.

### 3.3. Feature Selection and Model Development

All NC and AP features had ICC values > 0.75. Thirteen delta features had ICC values < 0.75 and, thus, were excluded. Subsequently, 55, 48, 110, and 19 redundant features were excluded from the NC, AP, NC + AP, and delta models, respectively. The MSEs of the NC, AP, NC + AP, and delta LASSO model in cross-validation were 0.154, 0.150, 0.149, and 0.151, respectively. After performing the LASSO regression, two, five, four, and six non-zero-weighted features were used to train the NC, AP, NC + AP, and delta models, respectively.

The selected features and the Gini importance in each decision tree model are listed in Table 3. In the NC model, “cluster tendency” and “cluster shade” contributed to the classification. In the AP model, “cluster shade”, “90th intensity percentile”, and “low-dependence low-gray level emphasis” contributed to the classification. In the NC + AP model, “cluster tendency”, “cluster shade”, “90th intensity percentile”, and “maximum intensity” contributed to the classification. In the delta model, “delta maximum intensity”, “delta discretized intensity uniformity”, “delta 90th intensity percentile”, “delta zone size entropy”, and “delta intensity range” contributed to the classification. The decision tree structures are shown in Supplementary Figures S1–S4. The models and code examples for using the models can be found in online files (https://github.com/DOCT-Y/TKI-RCC-delta-radiomics accessed on 21 December 2022).

### 3.4. Model Evaluation

The model performance of the training and validation cohorts is presented in Table 4. In the training cohort, the NC, AP, NC + AP, and delta models achieved AUC values of 0.849 (95% confidence interval [CI]: 0.779‒0.918), 0.872 (95% CI: 0.795‒0.949), 0.905 (95% CI: 0.853‒0.958), and 0.940 (95% CI: 0.890‒0.990), respectively. The AUC of the delta model was significantly different from that of the NC model (*p* = 0.049) but comparable to that of the AP and NC + AP models (*p* = 0.131 and 0.339, respectively) in the training cohort. The NC + AP model exhibited the highest sensitivity (0.882), and the delta model exhibited the highest accuracy (0.879).

In the validation cohort, the four models achieved AUC values of 0.710 (95% CI: 0.529‒0.891), 0.803 (95% CI: 0.677‒0.929), 0.708 (95% CI: 0.516‒0.900), and 0.916 (95% CI: 0.828‒1.000), respectively. The AUC of the delta model was significantly different from that of the AP and NC + AP models (*p* = 0.041 and 0.020, respectively) but comparable to that of the NC model (*p* = 0.056) in the validation cohort. The delta model exhibited the highest sensitivity (0.714) and accuracy (0.875).

**Figure 3:**
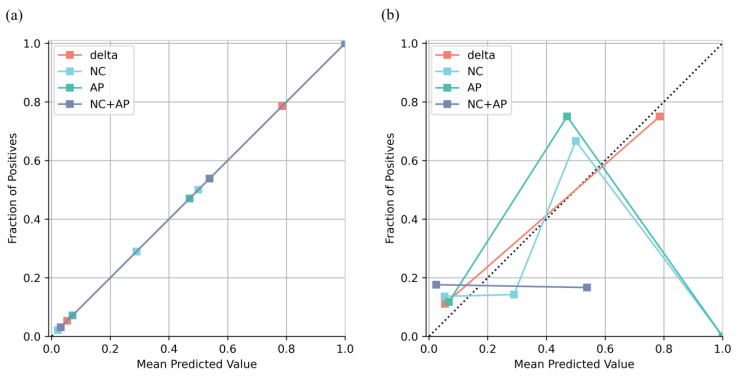
Calibration curves of each model in training (**a**) and validation (**b**) cohorts.

**Figure 4:**
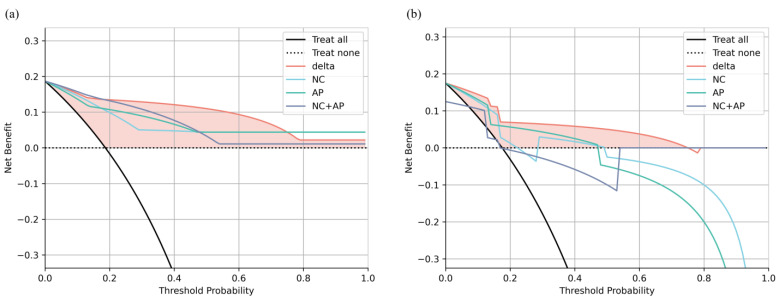
Decision curves of each model in training (**a**) and validation (**b**) cohorts. The filled area demonstrates the area between decision curve of the delta model, treat-none criteria, and treat-all criteria.

All models were well calibrated in the training cohort, whereas only the delta model remained well calibrated in the validation cohort (Figure 3). The result of the DCA showed that the net benefit of the delta model was greater than that of the NC, AP, and NC + AP models, as well as the treat-all and treat-none criteria (Figure 4).

## 4. Discussion

In this study, radiomics models based on pretreatment CT images were developed and internally validated to predict lesion-level short-term responses to TKIs in patients with advanced RCC. As a result, the model based on delta radiomics features exhibited the highest AUC value of 0.916 in the validation cohort. Its calibration curve and decision curve revealed good fitness and higher benefits in clinical practice. Therefore, the model derived from CT-based delta radiomics features could help predict lesion-level short-term response to TKIs to aid in defining treatment strategies and making clinical decisions in patients with advanced RCC.

In our study, the first tree node of the delta model correctly ruled out most of the SLs/RLs by using a larger delta intensity maximum value. This feature represented the largest difference in voxel intensities between the AP and the precontrast phase. The delta intensity maximum had the highest Gini importance in the delta model, which supported the hypothesis that the perfusion of lesions can predict the response to TKIs. In the following tree nodes, we found that some SLs/RLs showed lower values of delta-discretized intensity uniformity and delta 90th intensity percentile than PLs. The more homogeneous enhancement the lesion shows, the lower the number of gray levels in the AP the lesion has, and the larger the delta-discretized intensity uniformity is supposed to be. Similar to the delta intensity maximum, the delta 90th intensity percentile represented almost the largest difference in voxel intensities between the AP and the precontrast phase. Taken together, the lower values of the two features indicated that some SLs/RLs had lower enhancement than PLs, which contradicted the hypothesis. Thus, lesions with high enhancement may usually show PR or may remain stable, whereas the responses of the lesions with low enhancement are uncertain. This was reflected in the model performance: a high NPV value but a relatively low PPV.

Since TKIs target the VEGF pathway, lesion enhancement is, theoretically, a good predictor of response. However, lesions at different locations could not achieve the highest enhancement together at the scanning time point. The image intensity may vary among different vendors and scanners. Thus, the difference between the AP and the precontrast phase may not represent the true perfusion of the lesions. We noticed that the texture features, including cluster shade, cluster tendency, zone size entropy, and low-dependence low-gray-level emphasis, were predictive in the radiomics models. This result suggests that intratumor heterogeneity, in addition to perfusion, may reflect tumor aggressiveness and is valuable in the prediction of TKI response.

Previous studies used the average feature values of all the lesions in each patient to predict patient-level response to TKIs [3,4]. A recent study used AP radiomic features of a CT image to predict treatment response to TKIs [9]. The model did not achieve the best performance (AUC = 0.66). In our study, the AP model was also not the best, indicating that when predicting TKI response, AP features may not be the best solution. Our results showed the delta features, arithmetic differences between the NC and AP features, can better predict the early response of TKI treatment. Yet, studies on lesion-level prediction are scarce. In our study, radiomics models were developed based on lesion-level radiomics features, which were used to predict the response of each lesion. Heterogeneous radiological responses exist. The results of our study showed that heterogeneous responses to TKIs were observed in 13 of the 36 included patients (36.1%). Lesion-level prediction of the response to TKIs can help identify the lesions that may or may not benefit from the treatment. Metastasectomy is a potential treatment option, in addition to systemic therapy, to decrease tumor burden and sometimes achieve complete remission of the disease [10,11]. The surgical excision of metastases was reported to prolong the survival time of patients with mRCC [12,13,14]. Through stratification using the lesion-level radiomics predictive model, metastasectomy can be performed on some lesions with a high risk of progression, while the majority of lesions with a model-predicted preferable response can remain on TKI therapy.

This study has some limitations that must be acknowledged. First, selection bias inevitably existed because of the retrospective nature of the study. Second, the sample size and the number of PLs were limited. To reduce the risk of overfitting, we extracted only 107 radiomics features from the original CT images and did not use image filters. In addition, we fully reported the model performance of discrimination, calibration, and clinical utility to avoid optimism in the evaluation. Third, we could not assess the lesion-level PFS and, thus, could not perform the survival analysis. All the lesions in one patient were “lost” during the follow-up after switching the therapy owing to patient-level disease progression, including an increase in the sum of the longest diameters and the occurrence of new lesions. Thus, the current model predicted the response at a single time point. In addition, acquired resistance may develop as the treatment progresses. Thus, we developed the model for the prediction of response six months after treatment onset.

## 5. Conclusions

Models based on CT delta radiomics features may help predict the short-term response to TKIs in patients with advanced RCC and aid in lesion stratification for potential treatments.

## Figures and Tables

**Table 1:** Patient characteristics.

Parameters	PR (*n* = 14)	SD (*n* = 14)	PD (*n* = 8)
Age (years) *	55 (50–67)	58 (49–62)	62 (56–70)
Gender			
male	10 (71.4%)	9 (64.3%)	7 (87.5%)
female	4 (28.6%)	5 (35.7%)	1 (87.5%)
MSKCC group			
favorable risk	4 (28.6%)	3 (21.4%)	0 (0.0%)
intermediate risk	9 (64.3%)	7 (50.0%)	7 (87.5%)
poor risk	1 (7.1%)	4 (28.6%)	1 (12.5%)
IMDC group			
favorable risk	4 (28.6%)	3 (21.4%)	0 (0.0%)
intermediate risk	8 (57.1%)	5 (35.7%)	5 (62.5%)
poor risk	2 (14.3%)	6 (42.9%)	3 (37.5%)
Medication			
sunitinib	11 (78.6%)	6 (42.9%)	5 (62.5%)
axitinib	3 (21.4%)	7 (50.0%)	3 (37.5%)
sorafenib	0 (0.0%)	1 (7.1%)	0 (0.0%)
Lesion-level response			
homogeneity	8 (57.1%)	11 (78.6%)	4 (50.0%)
heterogeneity	6 (42.9%)	3 (21.4%)	4 (50.0%)

* The data are median with interquartile range in parentheses. Note: IMDC, International Metastatic Renal Cell Carcinoma Database Consortium score; MSKCC, Memorial Sloan Kettering Cancer Center score; PD, progressive disease; PR, partial response; SD, stable disease.

**Table 2:** Lesion Characteristics.

Parameters	Training Cohort (*n* = 91)	Validation Cohort (*n* = 40)	*p*-Value
Baseline size (mm) *	15.8 (11.8–23.2)	15.7 (13.2–20.0)	0.759
Six-month size (mm) *	12.9 (7.8–20.7)	14.1 (9.2–19.7)	0.747
Response			0.591
responding lesion	41 (45.1%)	15 (37.5%)	
stable lesion	33 (36.3%)	18 (45.0%)	
progressing lesion	17 (18.7%)	7 (17.5%)	
Location			0.165
lung	63 (69.2%)	32 (80.0%)	
kidney	10 (11.0%)	5 (12.5%)	
lymph node	5 (5.5%)	0 (0.0%)	
adrenal gland	3 (3.3%)	0 (0.0%)	
pancreas	3 (3.3%)	0 (0.0%)	
peritoneum	3 (3.3%)	1 (2.5%)	
pleura	2 (2.2%)	0 (0.0%)	
liver	2 (2.2%)	0 (0.0%)	
bone	0 (0.0%)	2 (5.0%)	

* The data are median with interquartile range in parentheses.

**Table 3:** Feature Importance in Each Model.

Model	Phase	Feature Class	Feature Name	Gini Importance
NC	noncontrast	GLCM	cluster tendency	0.783
	noncontrast	GLCM	cluster shade	0.217
AP	arterial phase	GLCM	cluster shade	0.409
	arterial phase	Intensity	90th intensity percentile	0.343
	arterial phase	NGLDM	LDLGLE	0.249
	arterial phase	Intensity	intensity kurtosis	0
	arterial phase	Intensity	maximum intensity	0
NC + AP	noncontrast	GLCM	cluster tendency	0.379
	noncontrast	GLCM	cluster shade	0.351
	arterial phase	Intensity	90th intensity percentile	0.245
	arterial phase	Intensity	maximum intensity	0.026
delta	/	Intensity	maximum intensity	0.332
	/	Intensity	discretized intensity uniformity	0.255
	/	Intensity	90th intensity percentile	0.201
	/	GLSZM	zone size entropy	0.122
	/	Intensity	intensity range	0.090
	/	Intensity	discretized intensity entropy	0

Note: AP, arterial-phase model; GLCM, gray-level co-occurrence matrix; GLSZM, gray-level size zone matrix; LDLGLE, low-dependence low gray-level emphasis; NC, noncontrast model; NC + AP, noncontrast plus arterial-phase model.

**Table 4:** Model Performance.

Model	Cut-Off		Sensitivity	Specificity	PPV	NPV	Accuracy	AUC (95% CI)	*p*-Value *
NC	0.290	Training	0.294	0.987	0.833	0.859	0.857	0.849 (0.779‒0.918)	0.049
		Validation	0.286	0.939	0.500	0.861	0.825	0.710 (0.529‒0.891)	0.056
AP	0.136	Training	0.706	0.878	0.572	0.929	0.846	0.872 (0.795‒0.949)	0.131
		Validation	0.429	0.909	0.500	0.882	0.825	0.803 (0.677‒0.929)	0.041
NC + AP	0.167	Training	0.882	0.838	0.556	0.969	0.846	0.905 (0.853‒0.958)	0.340
		Validation	0.143	0.849	0.167	0.824	0.725	0.708 (0.516‒0.900)	0.020
delta	0.131	Training	0.824	0.892	0.636	0.957	0.879	0.940 (0.890‒0.990)	/
		Validation	0.714	0.909	0.625	0.938	0.875	0.916 (0.828‒1.000)	/

* The Delong test for delta model and the others. Note: AP, arterial-phase model; AUC, area under receiver operating characteristic curve; CI, confidence interval; NC, noncontrast model; NC + AP, noncontrast plus arterial-phase model; NPV, negative predictive value; PPV, positive predictive value.

## Data Availability

The models generated during the current study are available in the GitHub repository (https://github.com/DOCT-Y/TKI-RCC-delta-radiomics accessed on 21 December 2022). The datasets analyzed during the current study are not publicly available due to privacy policies but are available from the corresponding author on reasonable request.

## Supplementary Materials

The following supporting information can be downloaded at: https://www.mdpi.com/article/10.3390/jcm12041301/s1, Figure S1: The tree structure of NC model; Figure S2: The tree structure of AP model; Figure S3: The tree structure of NC + AP model; Figure S4: The tree structure of delta model; Tripod-Checlist-Prediction-Model-Development; TRIPOD checklist; The checklist of TRIPOD to ensure the quality of reporting for predictive model studies.
