# Supplementary material for: Delta Radiomics Model Predicts Lesion-Level Responses to Tyrosine Kinase Inhibitors in Patients with Advanced Renal Cell Carcinoma: A Preliminary Result
Source: J Clin Med. 2023 Feb 6;12(4):1301. doi: 10.3390/jcm12041301 (PMC9966873; doi:10.3390/jcm12041301)
Supplement: Supplementary file 1 [file jcm-12-01301-s001.zip › jcm-2146211-supplementary.pdf]

The tree structure of NC model:

x[0]= CT_original_glcm_ClusterShade

x[1]= CT_original_glcm_ClusterTendency

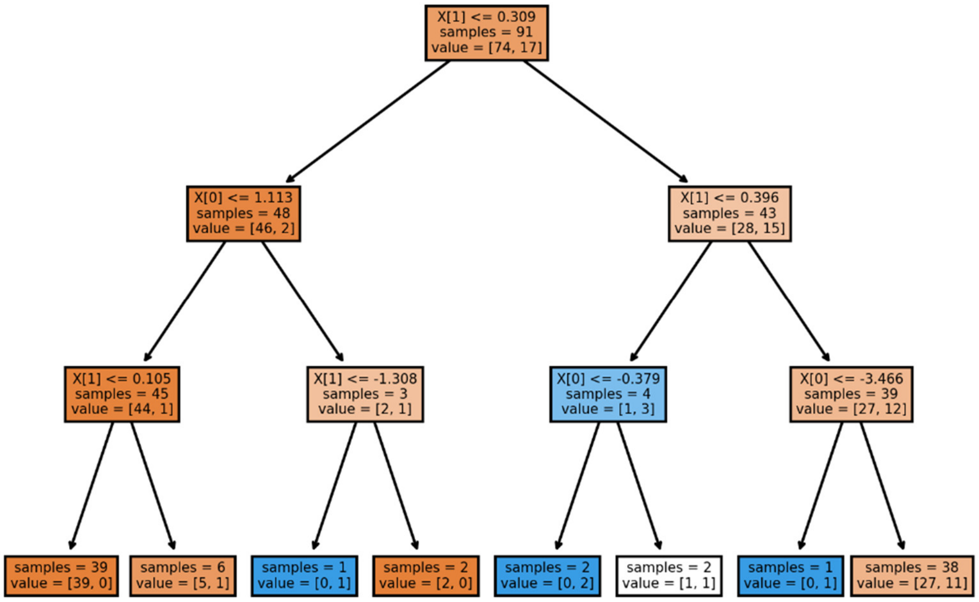

Figure S1. The tree structure of NC model.

The tree structure of AP model:

x[0]= CT_original_firstorder_90Percentile

x[3]= CT_original_glcm_ClusterShade

x[4]= CT_original_gldm_SmallDependenceLowGrayLevelEmphasis

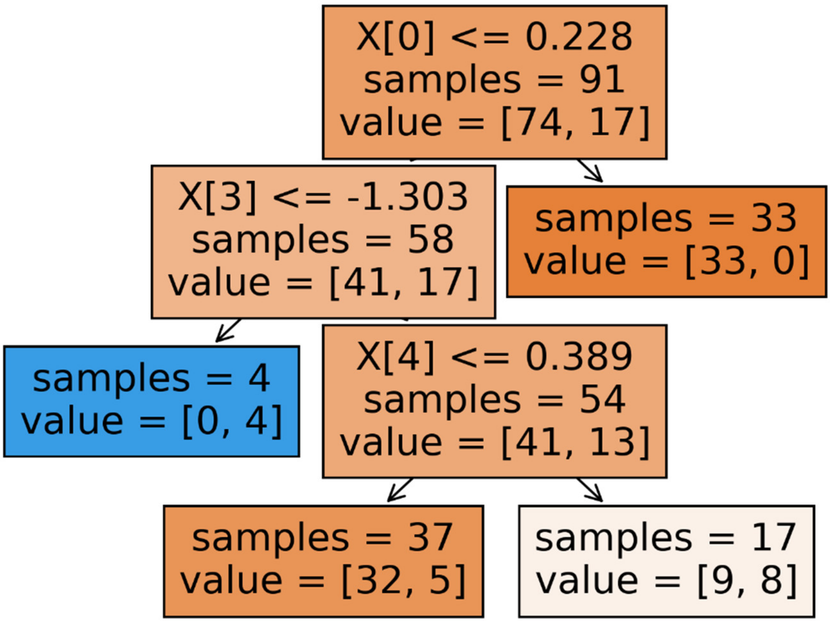

Figure S2. The tree structure of AP model.

The tree structure of NC+AP model:

x[0]= NCCT_original_glcm_ClusterShade

x[1]= NCCT_original_glcm_ClusterTendency

x[2]= APCT_original_firstorder_90Percentile

x[3]= APCT_original_firstorder_Maximum

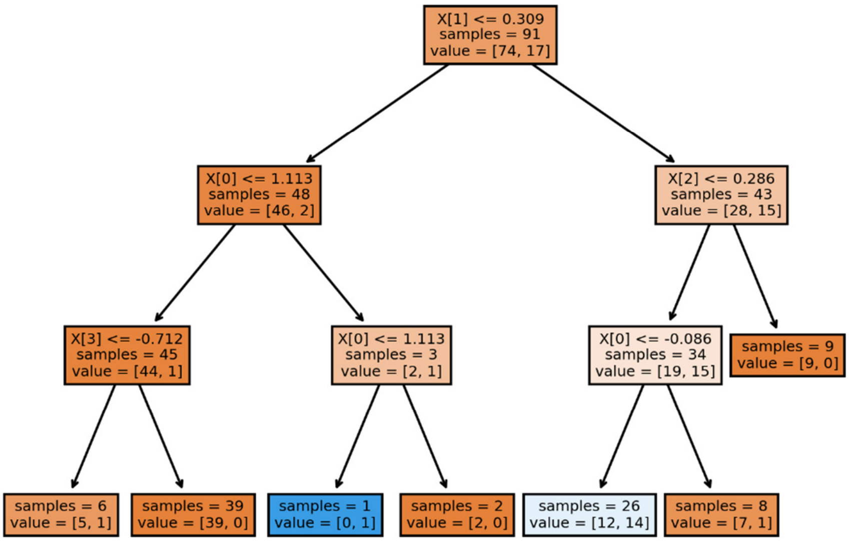

Figure S3. The tree structure of NC + AP model.

The tree structure of delta model:

x[0]= CT_original_firstorder_90Percentile

x[2]= CT_original_firstorder_Maximum

x[3]= CT_original_firstorder_Range

x[4]= CT_original_firstorder_Uniformity

x[5]= CT_original_glszm_ZoneEntropy

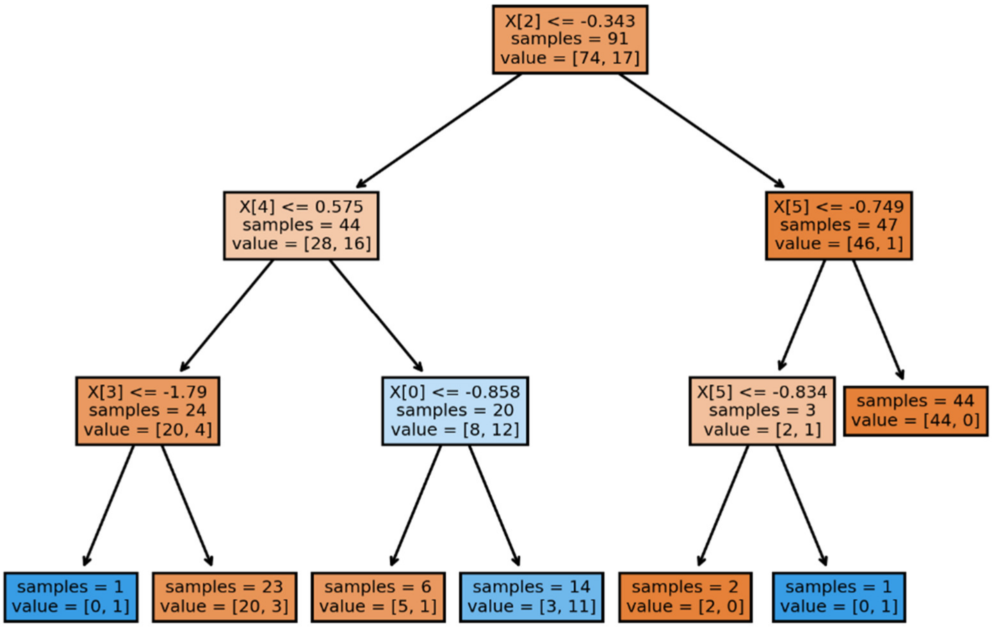

Figure S4. The tree structure of delta model.
